# Assessing the utility of frequency tagging for tracking memory-based reactivation of word representations

**DOI:** 10.1038/s41598-018-26091-3

**Published:** 2018-05-21

**Authors:** Ashley Glen Lewis, Herbert Schriefers, Marcel Bastiaansen, Jan-Mathijs Schoffelen

**Affiliations:** 1 Haskins Laboratories, 300 George Street, New Haven, CT 06510 USA; 2 Radboud University, Donders Institute for Brain, Cognition and Behaviour, Centre for Cognitive Neuroimaging, Nijmegen, The Netherlands; 3 Neurobiology of Language Department, Max Planck Institute for Psycholinguistics, Nijmegen, The Netherlands; 4 Radboud University, Donders Institute for Brain, Cognition and Behaviour, Centre for Cognition, Nijmegen, The Netherlands; 5 Tilburg University, Department of Cognitive Neuropsychology, Tilburg, The Netherlands; 6 NHTV University of Applied Sciences, Academy for Leisure, Breda, The Netherlands

## Abstract

Reinstatement of memory-related neural activity measured with high temporal precision potentially provides a useful index for real-time monitoring of the timing of activation of memory content during cognitive processing. The utility of such an index extends to any situation where one is interested in the (relative) timing of activation of different sources of information in memory, a paradigm case of which is tracking lexical activation during language processing. Essential for this approach is that memory reinstatement effects are robust, so that their absence (in the average) definitively indicates that no lexical activation is present. We used electroencephalography to test the robustness of a reported subsequent memory finding involving reinstatement of frequency-specific entrained oscillatory brain activity during subsequent recognition. Participants learned lists of words presented on a background flickering at either 6 or 15 Hz to entrain a steady-state brain response. Target words subsequently presented on a non-flickering background that were correctly identified as previously seen exhibited reinstatement effects at both entrainment frequencies. Reliability of these statistical inferences was however critically dependent on the approach used for multiple comparisons correction. We conclude that effects are not robust enough to be used as a reliable index of lexical activation during language processing.

## Introduction

Cognitive tasks require the coordinated activation of myriad different types of memory content. Such content is often represented at different levels of abstraction or hierarchical complexity^1^. Furthermore, these representations can be activated at different, often overlapping moments in time^1^. The ability to monitor precisely when, and the order in which, different sources of information are active in memory holds great promise for gaining deeper insight into the roles of these different types of information and how they interact during complex cognitive processing. For example, monitoring how quickly and in which order phonological, orthographic, and semantic information are activated during language production or speech comprehension can offer insights into the component processes of lexical retrieval, and inform cognitive and computational models designed to describe this complex cognitive process^2^.

One set of theories claim that for episodic memory, remembering entails the reinstatement of brain activity that was present when the memory was initially formed^3–7^. Neuroimaging work has provided support for such ‘memory reactivation’ accounts of remembering^8–16^. Memory reactivation effects in functional magnetic resonance imaging (fMRI) have the potential to be co-opted as an index of the activation of different types of memory content. These methods however offer relatively impoverished temporal resolution, making them poorly suited for investigating the precise timing of memory activation. A recent study instead used electroencephalography (EEG), and the well-known phenomenon of entrainment of frequency-specific steady-state oscillatory brain responses, to address exactly this timing question^17^.

In a typical subsequent memory paradigm, Wimber *et al*.^17^ presented participants with a list of target words to be learned during an encoding phase of their experiment, and following a short retention interval presented the same words along with new distractor words in a recognition phase. The key difference from previous studies was that during the encoding phase, words were presented on a background that flickered regularly at either 6 Hz or 10 Hz. Such flickering visual stimuli produce a steady-state visual evoked brain response in the EEG at the frequency of stimulation^18^. These frequency-specific entrainment effects are hypothesized to be incorporated as contextual information into the episodic memory representation participants form during encoding, so that memories can be ‘tagged’ with one of the two stimulation frequencies. This allowed Wimber *et al*.^17^ to show early reinstatement of this frequency-specific brain activity during the recognition phase (no background flicker), such that words presented during encoding on a background that flickered at 6 Hz resulted in greater phase consistency at 6 Hz in the EEG signal than words presented on a background that flickered at 10 Hz, and vice versa at 10 Hz. If this reinstatement can be demonstrated as a robust indicator of memory (re)activation, this would provide precisely the kind of tool described above for monitoring the timing and order of activation of different types of memory content during cognitive processing. For example, by first ‘tagging’ particular lexical items with a specific entrainment frequency during a memorization phase, we predict that it should be possible to monitor the time(s) at which these ‘frequency tags’ become reinstated during a subsequent sentence-reading task if the sentence contains that word (i.e., before, during, after the target word).

In the present study we perform a conceptual replication of Wimber *et al*.^17^ to examine the robustness of their memory reinstatement effects and establish the feasibility of using such a ‘frequency tagging’ approach as a means of monitoring the activation of memory content in real time. During the encoding phase of our subsequent memory experiment, participants were presented words to be memorized, and they had to indicate whether the word was concrete or abstract. After a retention interval, participants were presented these words (the target words) along with new distractor words and had to indicate how confident they were that the word had appeared during the encoding phase (C1-C6 corresponding to most confidently recognized down to most confidently not recognized). During the encoding (but not the recognition) phase, the background on which words were presented flickered regularly at either 6 or 15 Hz (the 6 Hz and the 15 Hz entrainment conditions, respectively). Such flickering visual stimuli produce a steady-state visual evoked brain response in the EEG at the frequency of stimulation^18^. Participants’ EEG was measured during both encoding and recognition phases of the experiment, allowing us to quantify the degree of frequency-specific phase consistency across trials in the EEG signal, by calculating inter-trial coherence^19^ (ITC). The difference in ITC at both 6 Hz and at 15 Hz between trials from the 6 Hz entrainment condition and trials from the 15 Hz entrainment condition was used to quantify whether a steady-state evoked response was present (encoding phase), and whether frequency-specific memory reinstatement occurred (recognition phase).

We hypothesized that if steady-state brain responses during encoding become associated with memory representations formed for memorized words, then during the recognition phase this frequency-specific activity should be reinstated and we should observe greater phase consistency in the EEG at 6 Hz for trials from the 6 Hz entrainment condition compared to trials from the 15 Hz entrainment condition, and vice versa at 15 Hz. Minimal criteria for establishing that these effects are robust were: (1) statistically significant memory reinstatement effects at both frequencies of interest (i.e., replication of the original findings); (2) statistical significance of the findings is not dependent on the particular approach used to correct for multiple statistical comparisons (a reasonable and appropriate alternative approach should not drastically change the outcome).

## Results and Discussion

**Figure 1:**
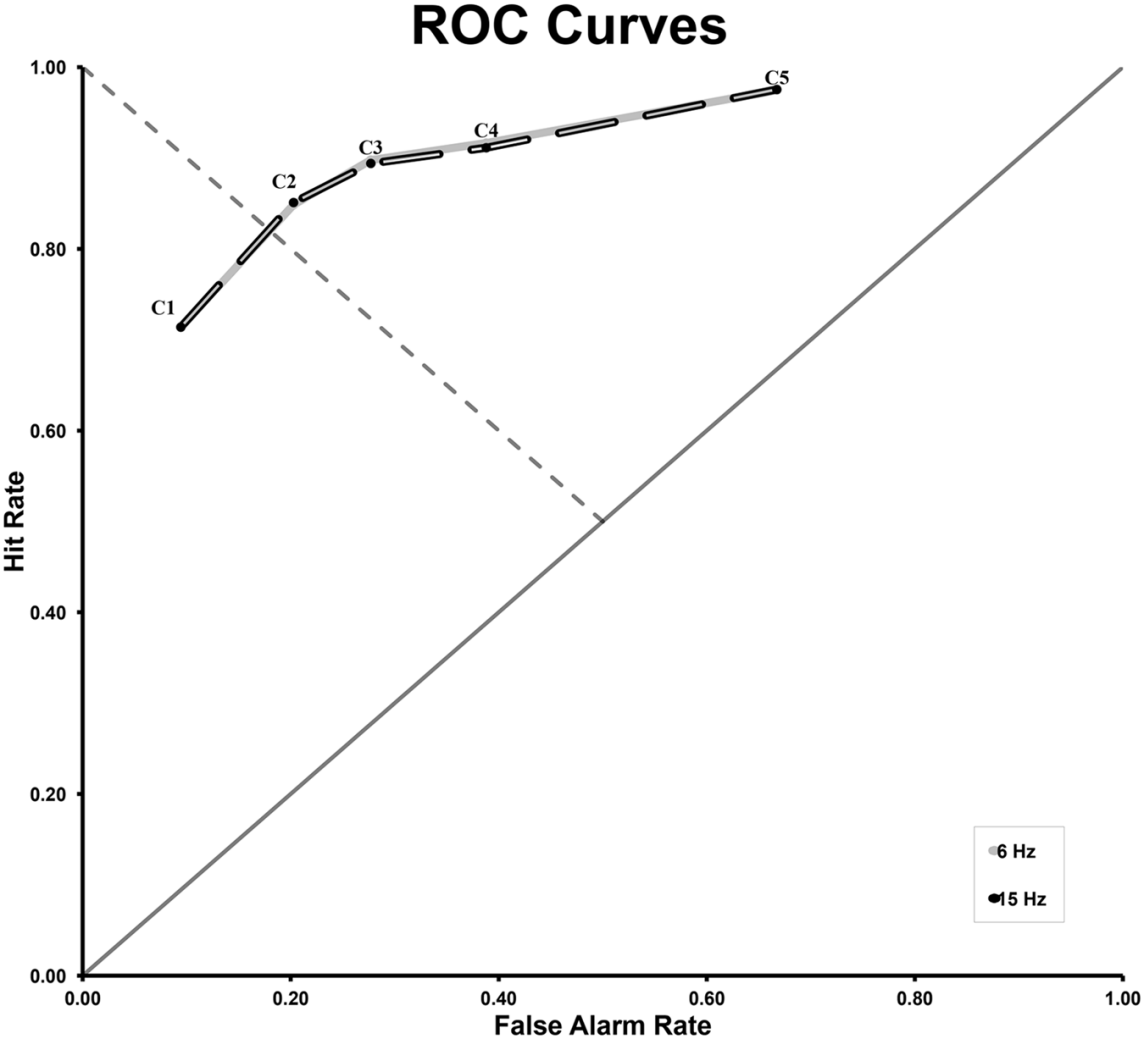
Receiver operating characteristic (ROC) curves for the 6 Hz and 15 Hz entrainment conditions. Cumulative hit rate is plotted against cumulative false alarm rate for different levels of confidence (C1-C6). Solid diagonal line = chance performance; dashed diagonal line = neutral response criterion.

Behavioural performance was not influenced by entrainment condition, as measured by recognition sensitivity (d′) and response bias (β). There were no statistically significant differences in recognition performance between the 6 Hz (d′_6Hz_: *M* = 1.99, SD = 0.64; β_6Hz_: *M* = 0.59, SD = 0.24) and the 15 Hz (d′_6Hz_: *M* = 1.95, SD = 0.55; β_6Hz_: *M* = 0.63, SD = 0.26) entrainment conditions (d′: t_20_ = 0.54, p = 0.6; β: t_20_ = −0.82, p = 0.42). Figure 1 shows overlapping ROC curves for the two entrainment conditions. For both entrainment conditions, the d′ results indicate that participants were clearly able to distinguish target from distractor words, and that there was a bias (β) toward responding that a word had already been seen during encoding (not surprising, since during recognition two thirds of trials comprised target words and only one third comprised distractor words).

**Figure 2:**
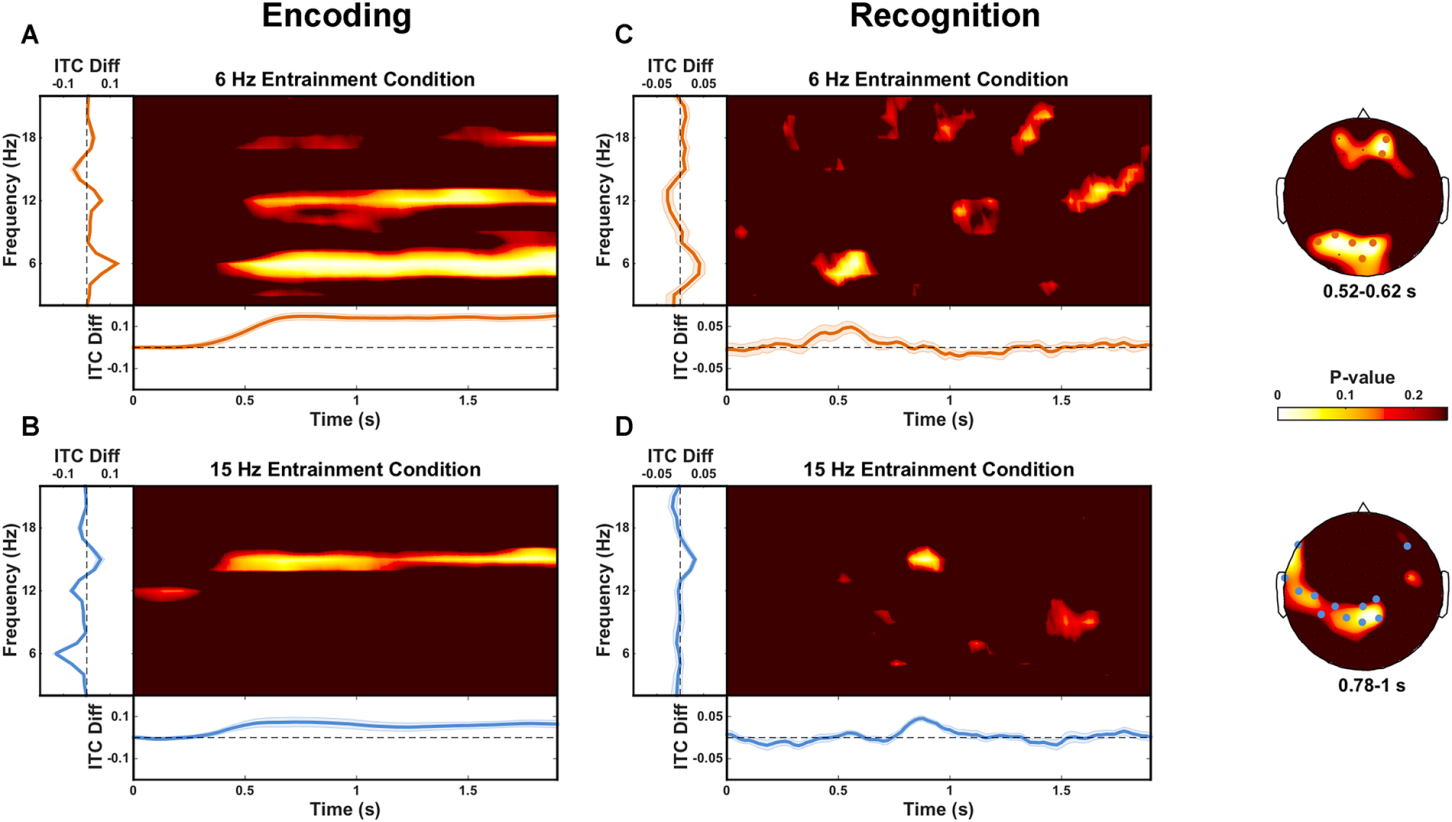
ITC differences during encoding and recognition. (**A** and **B**) Encoding: ITC difference between target words from the 6 compared to the 15 Hz entrainment condition (**A**) and target words from the 15 compared to the 6 Hz entrainment condition (**B**). Greater phase consistency was present at 6 Hz for words from the 6 Hz entrainment condition (340 to 1900 ms; 59 electrodes). Differences can also be observed at harmonic frequencies (12 Hz and 18 Hz). Similarly, greater phase consistency was present at 15 Hz for words from the 15 Hz entrainment condition (320 to 1900 ms; 51 electrodes). (**C** and **D**) Recognition: ITC difference between correctly recognized target words from the 6 compared to the 15 Hz entrainment condition (**C**) and for correctly recognized target words from the 15 compared to the 6 Hz entrainment condition (**D**). Greater phase consistency was present at 6 Hz for words from the 6 Hz entrainment condition, but this difference was only marginally significant after correcting for multiple comparisons (520 to 620 ms; 7 electrodes). Greater phase consistency was present at 15 Hz for words from the 15 Hz entrainment condition (780 to 1000 ms; 12 electrodes). In all panels, time-frequency plots show uncorrected P-values averaged over all electrodes exhibiting a statistically significant entrainment effect; line plots to the left show average ITC differences over the significant time interval and significant electrodes as a function of frequency; line plots at the bottom show average ITC differences over significant electrodes at the frequency of interest as a function of time; shaded regions indicate standard error of the mean; scalp plots (panels C and D) show uncorrected P-values averaged over the time interval of interest for the frequency of interest; electrodes showing differences over the entire time interval of interest are marked (orange = 6 Hz entrainment condition, blue = 15 Hz entrainment condition).

In order to investigate steady-state brain responses, we focused on evoked activity in the EEG, quantifying the degree of frequency-specific phase consistency across trials by calculating inter-trial coherence (ITC)^19^. The difference in ITC at both 6 Hz and at 15 Hz between trials from the 6 Hz entrainment condition and trials from the 15 Hz entrainment condition was used to quantify whether a steady-state evoked response was present (encoding), and whether frequency-specific memory reinstatement occurred (recognition). During encoding, entrainment was observed between about 320 and 1900 ms relative to word onset. Words from the 6 Hz entrainment condition exhibited stronger phase locking at 6 Hz than words from the 15 Hz entrainment condition (p_corr_ < 0.001) at all 59 electrodes (Fig. 2A). Similarly, words from the 15 Hz entrainment condition exhibited stronger phase locking at 15 Hz than words from the 6 Hz entrainment condition (p_corr_ < 0.001) at 51 of the 59 electrodes (Fig. 2B). For comparison, in addition to the original approach^17^ we employed a cluster-based correction^20^ for multiple statistical comparisons. This alternative was applied to data from both encoding and recognition, although our main interest was a comparison of the (potentially different) outcomes for recognition data. The cluster-based correction yielded very similar results for encoding, with entrainment between about 280 and 1900 ms relative to word onset (for cluster-based correction this is only an estimate based on electrode-time points contributing to the cluster that allowed us to reject our null hypothesis – no statistical claims about the extent of the effect should be drawn). Words from the 6 Hz entrainment condition exhibited stronger phase locking at 6 Hz than words from the 15 Hz entrainment condition (p_corr_ < 0.001) at all 59 electrodes. Similarly, words from the 15 Hz entrainment condition exhibited stronger phase locking at 15 Hz than words from the 6 Hz entrainment condition (p_corr_ < 0.001) at 58 of the 59 electrodes.

We were thus able to successfully entrain a steady state brain response at the frequency corresponding to the frequency of the flickering background during the encoding phase of our experiment. Furthermore, both approaches employed to correct for multiple statistical comparisons confirmed the effect and produced comparable results. Both studies thus show the presence of a frequency-specific steady-state oscillatory response at the entrainment frequency, and this has the potential to lead to the formation of an association between the oscillatory brain activity and a memory representation instantiated during encoding.

In order to test for memory-related reinstatement of frequency-specific entrainment during recognition, ITC differences at 6 and 15 Hz were compared between the two entrainment conditions. Only trials where target words were correctly recognized as having been presented during encoding (hit trials) were included in the statistical analyses. Reinstatement was observed in the 15 Hz entrainment condition between 780 and 1000 ms relative to word onset. Words from the 15 Hz entrainment condition exhibited stronger phase locking at 15 Hz than words from the 6 Hz entrainment condition (p_corr_ = 0.002) at 12 electrodes (Fig. 2D). There was only a marginally significant difference in phase locking at 6 Hz between words from the 6 Hz and the 15 Hz entrainment conditions (p_corr_ = 0.085), with 6 Hz reinstatement observed between 520 and 620 ms relative to word onset at 7 electrodes (Fig. 2C). The cluster-based approach for multiple-comparison correction yielded no statistically significant differences between the two entrainment conditions at either 6 Hz (p_corr_ = 0.904) or at 15 Hz (p_corr_ = 0.101).

We thus partially replicate the memory reinstatement effects observed by Wimber *et al*.^17^, but only definitively for one of our two entrainment conditions. On the other hand, both the statistically significant and marginal findings are sensitive to the approach used for correcting for multiple statistical comparisons, calling into question the strength and robustness of these reinstatement effects.

### ITC Bias

ITC is computed based on the consistency of the phase of the EEG signal across trials for a particular frequency. This reliance on phase makes it an inherently biased measure^21^, and as a result the expected ITC values are typically lower the larger the number of trials contributing to the computation of those values, even in the absence of true phase-locking. If this difference in bias is systematic across conditions (specifically, when there’s a systematic difference in the number of trials per condition across subjects), and if this bias is not accounted for in the statistical procedure, it could lead to incorrect statistical inference. For this reason, we were careful to ensure that trial numbers were comparable between the 6 Hz and the 15 Hz entrainment conditions across participants (see *Methods*). It is possible however that any within participant trial imbalances may have led to reduced sensitivity.

**Figure 3:**
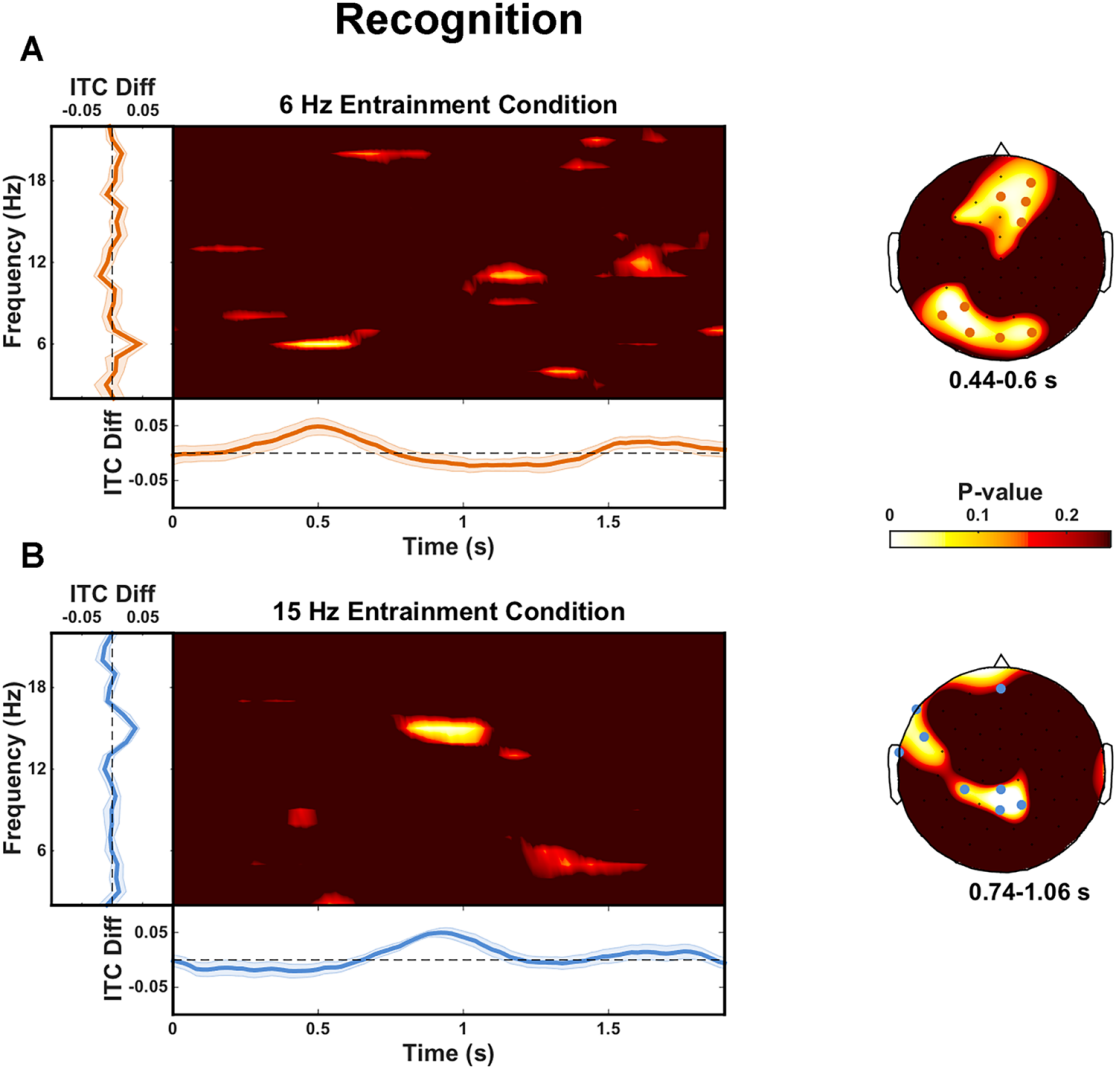
ITC differences during recognition: equal trial numbers within participants. ITC difference between correctly recognized target words from the 6 compared to the 15 Hz entrainment condition (**A**) and for correctly recognized target words from the 15 compared to the 6 Hz entrainment condition (**B**). Greater phase consistency was present at 6 Hz for words from the 6 Hz entrainment condition (440 to 600 ms; 9 electrodes). Greater phase consistency was present at 15 Hz for words from the 15 Hz entrainment condition (740 to 1060 ms; 8 electrodes). In all panels, time-frequency plots show uncorrected P-values averaged over all electrodes exhibiting a statistically significant entrainment effect; line plots to the left show average ITC differences over the significant time interval and significant electrodes as a function of frequency; line plots at the bottom show average ITC differences over significant electrodes at the frequency of interest as a function of time; shaded regions indicate standard error of the mean; scalp plots (panels A and B) show uncorrected P-values averaged over the time interval of interest for the frequency of interest; electrodes showing differences over the entire time interval of interest are marked (orange = 6 Hz entrainment condition, blue = 15 Hz entrainment condition).

In order to address this possibility, we ran the same analyses for data from the recognition phase of the experiment after equating the number of hit trials contributing to the ITC computation in each of the 6 and 15 Hz entrainment conditions (for details see *Supplementary Information*). Reinstatement was still present in the 15 Hz entrainment condition in roughly the same time interval as was initially observed (between 740 and 1060 ms relative to word onset). Words from the 15 Hz entrainment condition exhibited stronger phase locking at 15 Hz than words from the 6 Hz entrainment condition (p_corr_ = 0.021) at 8 electrodes (Fig. 3D). Moreover, the marginal difference in the 6 Hz entrainment condition in our initial analysis now exhibited a statistically significant reinstatement effect between 440 and 600 ms relative to word onset. There was a statistically significant difference in phase locking at 6 Hz between words from the 6 Hz and the 15 Hz entrainment conditions (p_corr_ = 0.004) at 9 electrodes (Fig. 3C). The cluster-based approach for multiple comparison correction yielded no statistically significant differences between the two entrainment conditions at either 6 Hz (p_corr_ = 0.559) or at 15 Hz (p_corr_ = 0.195).

This suggests that these reinstatement effects indeed appear to be sensitive to the relative number of trials contributing to the ITC values computed for each condition. After accounting for this difference, we successfully replicate the reinstatement effects reported in Wimber *et al*.^17^ in both entrainment conditions. The main difference appears to be that when trial numbers are equated within participants, more time points and more electrodes exhibit the consistent ITC difference at 6 Hz (Fig. 3C) than when trial numbers are not equated (Fig. 2C). Equating trial numbers within participants does not influence the outcome when employing the cluster-based permutation approach for multiple comparisons correction, and the strength and robustness of these reinstatement effects thus remain questionable. As in Wimber *et al*.^17^, our reinstatement effects during recognition were present at a subset of the electrodes that exhibited an entrainment effect during the encoding phase, although this is not surprising, since entrainment was observed at almost all scalp electrodes during encoding.

### Timing of Reinstatement

Interestingly, our memory reinstatement effect at 15 Hz occurs later (starting around 780 ms after target word onset; around 740 ms after target word onset when equating trial numbers per condition) than the effects reported by Wimber *et al*.^17^, where reinstatement in both their 6 and 10 Hz entrainment conditions occurred between 0 and 300 ms after target word onset. This is also the case for our effect at 6 Hz (starting around 520 ms after target word onset for the marginal result; around 440 ms after target word onset when equating trial numbers). One intriguing explanation is that because we employed a deep encoding task (rather than the shallow encoding task employed by Wimber *et al*.^17^) the ‘frequency tags’ became associated with a different aspect of the memory representation instantiated during encoding. A deep encoding task requires participants to attend to the semantic features of the words being memorized (e.g., judge whether a word falls within a particular semantic category or, as in our study, judge whether the word is concrete or abstract)^22^. A shallow encoding task on the other hand requires participants to attend only to the physical characteristics of the words (e.g., detect whether a particular letter forms part of the word). It is possible that when an association is formed between the frequency-specific entrainment and a memory trace during encoding, the type of information (word form vs meaning) within the memory trace with which the association is formed depends on whether a shallow or deep encoding task is employed. It is well established that for word recognition, semantic information is typically activated later than word form information during lexical retrieval^23^, which is in line with the fact that our reinstatement effects (deep encoding task) occur later than those reported in Wimber *et al*.^17^ (shallow encoding task). If this explanation proves correct, it has important implications for whether or not these memory reinstatement effects are indeed, as Wimber *et al*.^17^ suggested, an index of early ecphoric processing during memory retrieval^24,25^. Ecphory refers to early processing (preceding memory retrieval) responsible for prompting the activation of a stored memory trace. It is thought to be unconscious and to occur rapidly after a retrieval cue, suggesting that our relatively late reinstatement effects are unlikely to reflect the neural underpinnings of this stage of memory retrieval. Further investigation is clearly warranted.

Depth of encoding may indeed turn out to be an important difference between the two studies (see Table 1 for all key differences), since in the memory literature the overshadowing and outshining hypotheses both claim that contextual cues may be relied upon less at encoding and retrieval respectively when alternative non-contextual memory cues are present^26^. A focus on semantic aspects of the information being encoded into memory (as is required for deep encoding) may arguably provide such alternative (possibly more associative) cues and thus to some extent suppress the reinstatement of contextual (frequency specific entrainment) information during recognition. Precisely how this relates to our findings of later reinstatement of frequency-specific entrainment is unclear.

**Table 1:** Key differences between designs of Wimber *et al*. and present study.

*Factor*	*Wimber et al*.	*Lewis et al*.
Encoding task	Shallow	Deep
Entrainment frequencies	6 and 10 Hz	6 and 15 Hz
No. ‘Old’ words (recognition)	240	200
No. ‘New’ words (recognition)	120	100
Task during retention period	Unclear	Flanker task
Offline filtering	Unclear	Band-stop: 50,100,150 Hz (line noise) Low-pass: 30 Hz
EOG rejection/correction	Unclear	ICA
Spectral decomposition	Gabor transform	Short-time Fourier transform with Hanning tapered sliding window
Temporal precision	Unclear	1 Hz
Spectral precision	Unclear	1 s

Unclear indicates either that this factor was not reported or that it could not be adequately determined based on what was reported.

Another difference between the studies was in the degree of temporal smoothing used for the ITC calculation. We chose a relatively large degree of temporal smoothing in order to obtain a frequency precision of 1 Hz, and in order to capture an integer number of periodic cycles of the 6 Hz oscillatory entrainment. In relation to this point, two aspects of the analyses in Wimber *et al*.^17^ are unclear. First, the reported details of their spectral decomposition were not sufficient to establish the temporal and spectral precision achieved in their study. These details are important because improved temporal precision (at the expense of spectral precision) might be more sensitive to detecting more transient reinstatement effects, but it then becomes questionable whether or not what is detected constitutes reinstatement that is specific to the frequency range of interest. Second, the method by which the time windows of interest to be entered into the statistical analyses for both the encoding (0 to 2000 ms relative to word onset) and recognition (0 to 300 ms relative to word onset) phases of their experiment were selected was not reported in Wimber *et al*.^17^ (neither in their supplementary materials). Nor was it possible to use the exact same method by which this selection was made, by looking into the literature cited in support of the statistical method they employed. To address each of these uncertainties we performed some additional exploratory analyses, the results of which are presented below.

To address the inherent time-frequency trade-off we ran the same analyses for data from the recognition phase of our experiment, but this time with a 500 ms sliding time window (instead of the 1000 ms window we initially employed) used for spectral decomposition. We selected a 500 ms window because inspection of Fig. 2 from Wimber *et al*.^17^ suggests that their settings probably achieved frequency precision of approximately 2 Hz. Results were largely unchanged (see *Supplementary Information* for details), with reinstatement again observed at both 15 and 6 Hz (still marginal at 6 Hz) around the same time (between 780 and 1000 ms and between 520 and 620 ms relative to word onset respectively) and at the same number of electrodes (12 and 7 electrodes respectively), as in our initial analyses (see Fig. S1). We conclude that choice of temporal smoothing (within reasonable parameter settings based on the data and the experimental question) does not appear to affect our ability to detect memory reinstatement effects in our data.

To address the uncertainty about selection of a time window of interest we investigated a couple of plausible alternatives. Both analyses were run with trial numbers equated between the two entrainment conditions within participants. One possibility is that instead of averaging over some time window of interest, Wimber *et al*.^17^ took the maximum number of electrodes across all time points from 0 to 2000 ms exhibiting a statistically significant effect (uncorrected) as their metric for constructing a permutation distribution to determine how unlikely the observed maximum number of electrodes exhibiting an effect was to have occurred by chance. In this case the 0 to 300 ms time window of interest reported would correspond to a time window over which the relevant maximum number of electrodes exhibiting an effect was sustained. Employing such an approach with our data did not produce statistically significant results for either the 6 Hz (p_corr_ = 0.353) or the 15 Hz (p_corr_ = 0.143) entrainment condition. The other plausible alternative is that the 0 to 300 ms time window was simply selected a priori (although this was never mentioned in Wimber *et al*.^17^) based on the hypothesis that contextual reinstatement effects were expected to occur early in time. Employing this approach with our data did not produce statistically significant results for either the 6 Hz or the 15 Hz entrainment condition (there were no time points exhibiting effects for at least 4 electrodes).

Notably, both our 15 Hz and our marginal 6 Hz reinstatement effects are present over largely spatially contiguous electrodes (either one or two clusters). The effect in Wimber *et al*.^17^ at 6 Hz is present over spatially non-contiguous electrodes (see their Fig. 2C), with only their 10 Hz reinstatement effect showing some spatial contiguity over left frontocentral electrodes. While the spatial distribution of memory reinstatement effects is likely related to the network of regions that were active during encoding^13,14^, there should still be a large degree of spatial contiguity when observing EEG activity at the scalp due to volume conduction^27^. It is certainly possible that spatial contiguity of genuine effects may be obscured to some extent by statistical thresholding, but electrodes exceeding the threshold are still likely to exhibit a tendency to cluster together rather than exhibit a widely distributed and disconnected pattern. Future work should consider attempting to reconstruct the cortical sources of these memory reinstatement effects in order to investigate whether it does indeed constitute reactivation of neural activity in the same brain regions or networks that were active during entrainment.

## Concluding Remarks

The primary goal of our study was to investigate whether or not a ‘frequency tagging’ and subsequent memory reinstatement approach could be used to investigate the timing of activation of lexical information during language comprehension. One important prerequisite is that the memory reinstatement effects are robust so that their presence, and more importantly their absence, can be taken as an indication of whether or not lexical information is activated at a particular moment. To be clear, we do not require that reinstatement can be observed robustly on a single-trial basis, only that it be robust in the mean neural response over trials for a particular entrainment condition across participants. Our results suggest that this prerequisite is not met, as we show a reliable memory-related reinstatement effect only at 15 Hz but not at 6 Hz, despite comparable subsequent memory performance based on the behavioural measures of sensitivity and response bias. While we do observe a statistically significant reinstatement effect at 6 Hz after equating trial numbers for each condition within participants, the ability to report an effect appears to be highly sensitive to alternative approaches to multiple comparisons correction. The cluster-based random permutation approach^20^ has proven a useful procedure for controlling the family-wise error rate based on physiologically plausible assumptions. We acknowledge however that different statistical procedures are sensitive to (and therefore could be more appropriate for investigating) different aspects of the data^28^. Effects in both the 6 Hz and the 15 Hz entrainment conditions (after equating trial numbers) appear to form at least 2 spatially contiguous clusters, and this potentially reduces the sensitivity of the cluster-based procedure for detecting potential effects. The approach employed by Wimber *et al*.^17^ is likely to be more sensitive to detecting effects at electrodes that do not form spatially contiguous clusters, which is certainly physiologically plausible after statistical thresholding. On the other hand, this approach also needs to deal with the temporal dimension of the data, and the way this is handled critically seems to determine the inferential conclusions that can be drawn. On this basis, we think it is unclear whether the effects we report at 15 Hz and at 6 Hz (with equated trial numbers) are reliable and robust, and therefore suggest that the issue of how best to characterize such memory reinstatement effects warrants further investigation.

We cannot rule out that some of the minor changes to our experiment (see Table 1) caused our memory-related reinstatement effects to be less clear-cut than those reported by Wimber *et al*.^17^. Nevertheless, further investigation is needed before it can be concluded that these entrainment effects can be used as an adequate marker for tracking the moment-by-moment activation of memory content during cognitive processing. In precisely this context future studies should consider employing Bayesian statistical approaches^29^ that allow one to explicitly quantify the evidence for the alternative hypothesis. This could potentially provide a more sensitive measure of whether or not memory reinstatement is present at a specified time, along with additional information about the strength of that activation.

## Methods

### Participants

Twenty-eight native speakers of Dutch took part in the experiment, 21 of whom were included in the final analysis (8 males, 13 females; aged 19 to 36). Participants provided informed consent and were paid or equivalently rewarded with course credits for their participation. All participants reported normal or corrected-to-normal vision and were right handed. None of the participants reported any neurological impairment. The study was approved by the local ethics committee (CMO – Commissie Mensgebonden Onderzoek – Arnhem-Nijmegen region) and carried out in accordance with the guidelines laid out in the Declaration of Helsinki.

Three participants were excluded from the final analysis due to recording problems. Four other participants were excluded due to poor data quality, which resulted in too few trials for the ITC analysis (fewer than 35 hit trials in either of the entrainment conditions during the recognition period).

### Stimulus Materials

Target stimuli consisted of 200 Dutch words selected from a large database of Dutch-English translation pairs^30^. Words were selected from this database because we planned to also use the English translations of the Dutch words in follow up experiments. Approximately two thirds of the words (67.5%) referred to concrete entities (e.g., *table* or *carrot*), while a third (32.5%) referred to abstract concepts (e.g., *revenge* or *wisdom*).

A total of 100 Dutch words were selected as distractor items to be used during the recognition phase of the experiment. Every participant saw all 200 target and 100 distractor stimuli over the course of the experiment. Which target and distractor items were seen in the first and second blocks of the experiment, as well as which half of the distractor items was presented together with which half of the target items in any single block, was counterbalanced across participants (see below for description of experimental blocks). Whether a target word was presented on a background flickering at 6 or at 15 Hz was also counterbalanced across participants. Resulting experimental lists for each block were then pseudorandomized according to the following criteria (the same for both encoding and recognition phase experimental lists): (1) no more than three consecutive repetitions of concrete or abstract words; (2) words from the same semantic category appeared at least three experimental items apart; (3) no more than two consecutive repetitions of a word presented with the same frequency of flickering background during the encoding phase (6 Hz or 15 Hz).

During the retention interval participants performed a flanker task. Stimuli for this task consisted of a central arrow pointing either to the left or to the right of the display. These were ‘flanked’ to both the left and the right by either three arrows or three equals signs (neutral condition). The flanker arrows could either point in the same direction as the central arrow (congruent condition) or in the opposite direction to the central arrow (incongruent condition). This task was only used to ensure that participants did not have time for rehearsal of the learned words during the retention interval, and thus these data were not analysed.

### Design and Procedure

Participants were tested in a dimly lit, sound-attenuating and electrically shielded booth. They were seated comfortably in front of an LCD computer monitor, with a viewing distance of between 70 and 80 cm. Words were presented in white on a rectangular black box using a 20-point sized uppercase Consolas font type. All words subtended a visual angle of 3.13° vertically. The black box measured 350 × 300 dpi and was presented centrally on a dark grey background encompassing the remainder of the display (1920 × 1080 dpi).

The experiment consisted of two blocks, each comprised of three experimental phases. First, in an encoding phase, participants learned 100 Dutch words while judging whether the words presented were concrete or abstract. We used a ‘deep encoding’ task rather than the ‘shallow encoding’ task used in Wimber *et al*.^17^. In principle, this should not make any difference to the memory reinstatement effects, and since we planned to use the present data as a basis for follow up experiments targeting the semantics of the words, we decided to draw participants’ attention to semantic information by using a deep encoding task. Directly following the encoding phase participants completed a flanker task (90 trials: 30 congruent; 30 incongruent; 30 neutral) that was unrelated to our experimental manipulation. This was used to ensure that participants did not have the opportunity to rehearse learned words during this 5- to 6-minute retention interval. Finally, in the recognition phase of the experiment participants saw target words they had learned during the encoding phase for that block, interspersed with 50 new Dutch distractor words they had not seen during encoding. They were required to judge whether or not these were words they had seen during encoding, and to indicate how certain they were of their response. Before the start of the first block participants completed a short practice session with items not used in the main experiment in order to ensure that they understood the task for each phase. Each block lasted approximately 40 minutes, with the entire experiment (including preparation and practice) taking around 2 hours.

During the encoding phase of the experiment, each trial began with the presentation in the centre of the screen of three asterisks two spaces apart for 2000 ms, indicating that participants could move their eyes and blink. This was immediately followed by a fixation cross presented in the centre of the screen for between 1000 and 1750 ms, indicating that eye movements and blinking should be avoided and the word was about to appear. Next, a word was presented in the centre of the screen for 2500 ms. During the presentation of the word the black background box flickered regularly at either 6 or 15 Hz, changing from black (RGB: 0, 0, 0) to the dark grey (RGB: 125, 125, 125) colour of the display background and back to black. Directly following the word, a question mark was presented in the centre of the screen for a maximum duration of 1500 ms, indicating that participants should provide a (subjective) judgment about whether the word referred to something abstract or concrete. The question mark disappeared immediately upon a response from the participant, and the next trial began. Participants were instructed to remember the words as they would be tested in the recognition phase of the experiment on whether or not they had seen them during the encoding phase. The subjective judgment about whether a word referred to something concrete or abstract was made by a button press with either their left or right index finger. For half of the participants the left index finger was used for words they thought were concrete and the right for words they thought were abstract, and vice versa for the other half of the participants.

During the recognition phase of the experiment, trials were the same as during the encoding phase, except that this time words were presented for 2000 ms and the black background box did not flicker. Participants were instructed to press a button with one hand in case they had seen the word during the encoding phase of the current block, or with the other hand in case it was a word they had not seen during the encoding phase. For each of these options participants had to indicate how certain they were about their response, with the index finger indicating complete certainty, the middle finger indicating medium certainty, and the ring finger indicating complete uncertainty. Response mapping was again counterbalanced across participants.

During the retention interval (the flanker task), each trial began with the presentation in the centre of the screen of three asterisks two spaces apart for between 1500 and 2500 ms, indicating that participants could move their eyes and blink. This was immediately followed by a fixation cross presented in the centre of the screen for 500 ms, indicating that eye movements and blinking should be avoided and the stimulus was about to appear. Next, the flanker stimuli (without the central target arrow) were presented in the centre of the screen for 100 ms, directly followed by the flanker stimuli with the central target arrow for 50 ms. Finally, a question mark was presented in the centre of the screen for a maximum duration of 1500 ms, indicating that participants should use their left or right index finger to press the button corresponding to the direction in which the central arrow was pointing. The question mark disappeared immediately upon a response from the participant, and the next trial began.

### EEG Recordings

**Figure 4:**
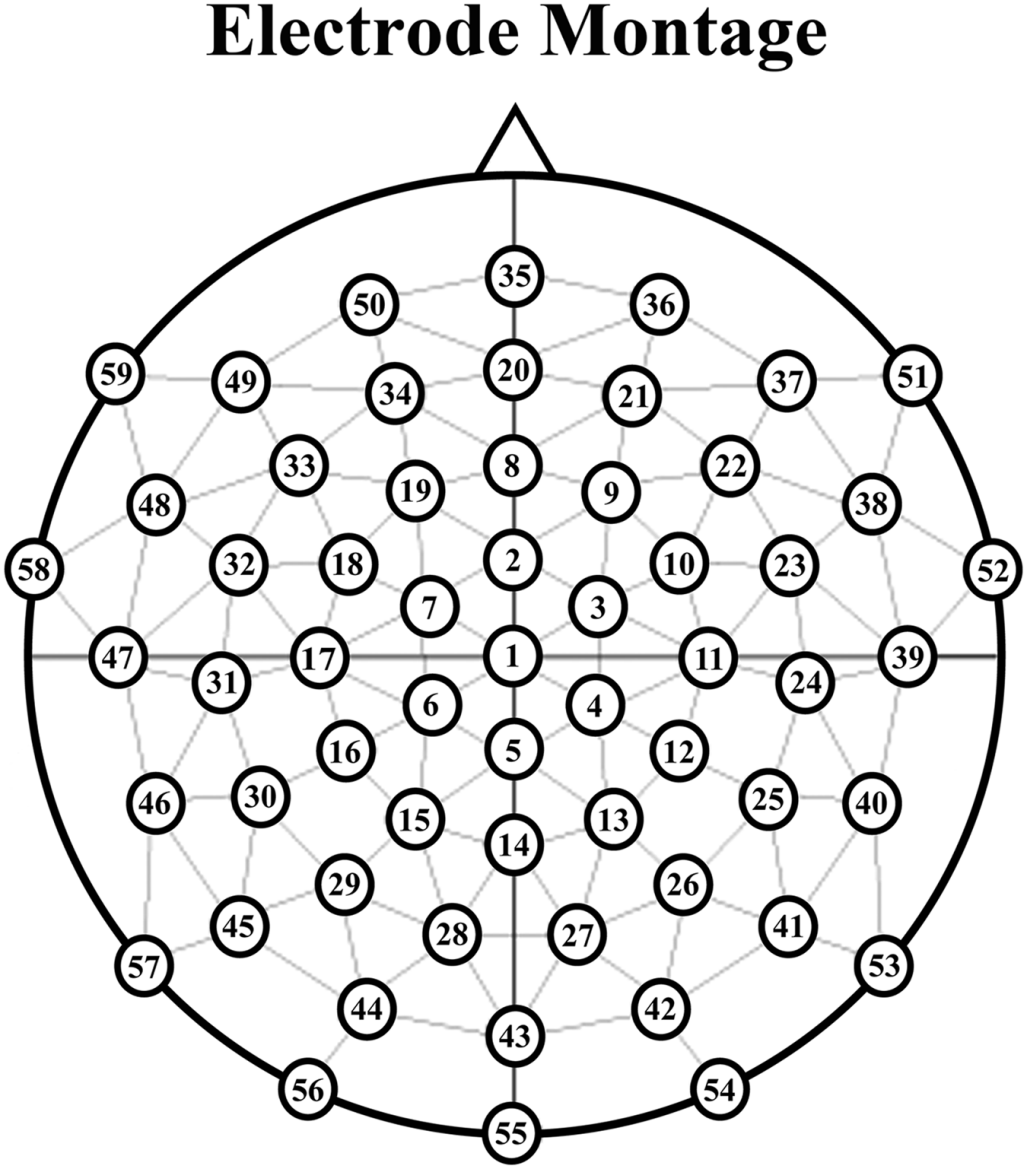
Positions of scalp electrodes in the EEG cap.

Participants were fitted with a 59 electrode cap with electrodes positioned in the geodesic arrangement shown in Fig. 4. EEG signals were recorded using 59 Ag/AgCl active sensors mounted in the cap and referred to the left mastoid. An additional electrode was placed on participants’ right mastoid, and a ground electrode was placed on the centre of the forehead. Another electrode was placed on the suborbital ridge of participants’ left eye for recording eye-blinks.

Electrode impedance was kept below 20 kΩ. EEG and EOG recordings were amplified using BrainAmp DC amplifiers (Brain Products GmbH, Gilching, Germany) with a band-pass filter of 0.016 to 200 Hz, digitized online with a sampling frequency of 500 Hz and stored for offline analysis.

### Behavioural Data Analysis

Recognition performance was assessed separately for the 6 Hz and 15 Hz entrainment conditions, using the signal detection measures recognition sensitivity (d′) and response bias (β), as well as by plotting individual (per participant) and mean receiver operating characteristic (ROC) curves^31,32^. For plotting the ROC curves, participants’ responses during the recognition phase of the experiment were ordered according to the level of certainty of their response from C1 (completely certain seen word during encoding phase) to C6 (completely certain haven’t seen word during encoding phase). The hit rate was calculated as the proportion of target words from the encoding phase correctly identified as previously seen, while the false alarm rate was calculated as the proportion of distractor words incorrectly identified as seen during the encoding phase. These scores were used to calculate recognition sensitivity and response bias for each participant. A paired-samples T-test was then used to assess whether there were any systematic differences across participants between the 6 Hz and 15 Hz entrainment conditions in either of these measures. Trials in which participants failed to make a response were excluded from the above analyses. This behavioural analysis is based only on data from participants who were included in the final EEG analysis.

### EEG Data Pre-processing

EEG data were analysed using the FieldTrip toolbox^33^ running in a Matlab environment (R2014b; Mathworks, Inc.). The following pre-processing steps were applied separately to the data from the encoding and the recognition phases of the experiment. For each participant, a band-stop filter was applied at 50, 100, and 150 Hz in order to minimize the effects of power line interference (50 Hz), and data were segmented from -1000 to 2500 ms relative to the onset of each word. The data were then visually inspected, and any electrodes exhibiting non-stationary artefacts in a large number of trials were removed from the data. Each electrode was then re-referenced to the average of all scalp electrodes (common average reference).

Next, the data were decomposed into independent components (ICA using the ‘runica’ implementation in FieldTrip with default settings). Components which captured eye-blinks or horizontal and vertical eye movements were removed and the remaining components were recombined^34,35^. Between 0 and 5 components were removed per participant. Electrodes that had been removed were then recovered based on the average activity at neighbouring electrodes, and a low-pass filter was applied at 30 Hz. Each data segment was then demeaned using the mean over the entire segment and any linear trends were removed. Any remaining artefact-containing trials were removed after visual inspection of all data segments.

Finally, the data were segmented into 6 Hz and 15 Hz entrainment conditions from −500 to 2500 ms relative to word onset. For the data from the recognition phase of the experiment, only data segments corresponding to hit trials (regardless of participants’ certainty rating) were included for further analysis. There were no statistically significant differences between the number of remaining trials in the two conditions for either the encoding (6 Hz: *M* = 61.95, SD = 7.66; 15 Hz: *M* = 64, SD = 8.29; p = 0.24) or the recognition (6 Hz hits: *M* = 56.81, SD = 11.16; 15 Hz hits: *M* = 57.43, SD = 8.93; p = 0.74) phase of the experiment.

### Inter-trial Coherence Analysis

Our analysis of the EEG data focused on the degree of phase consistency across trials relative to stimulus onset within a given frequency band. To that end, we computed the inter-trial coherence (ITC^19^; sometimes referred to as the phase-locking index^17^) for each participant from 0 ms to 2000 ms relative to the onset of each target word. For all trials of the encoding phase and hit trials of the recognition phase of the experiment, ITC was computed separately for the 6 Hz and 15 Hz entrainment conditions. First, time-resolved Fourier spectra of the data between 2 and 22 Hz were computed using a sliding window approach. Sliding windows of 1000 ms were applied in frequency steps of 1 Hz and time steps of 20 ms, and each window was tapered using a Hanning taper, to reduce spectral leakage. This resulted in a frequency resolution of 1 Hz, while the estimate at each time point is averaged data from the preceding and following 500 ms. Next, the Fourier spectrum of each trial was normalized by its amplitude, and the result was averaged across all trials for a particular entrainment condition. Taking the absolute value of this complex-valued average provided a frequency-resolved measure of the degree of trial-to-trial phase consistency over time (ITC).

### Statistical analyses

For data from both the encoding and recognition phases of the experiment, statistical significance was evaluated using the approach described by Wimber *et al*.^17^. In a first step, non-parametric Wilcoxon signed-rank tests were used to compare the 6 and 15 Hz entrainment conditions for every time-frequency pair at each electrode (p < 0.05 considered significant; one-tailed test). This resulted in uncorrected P-values for every time-frequency pair at each electrode. A time window of interest was then selected for data points at 6 Hz and at 15 Hz by selecting the largest time window exhibiting adjacent significant time points on at least 4 electrodes. The latter restriction was employed because physiologically meaningful effects tend to cluster at more than 1 electrode due to volume conduction (although admittedly after thresholding this may not necessarily be the case), and because in Wimber *et al*.^17^ when clustering was observed this was present for at least 4 electrodes (see their Fig. 2 for the 10 Hz condition). In a second step, a randomization approach (randomly permuting condition labels for each participant) was used to ensure that the number of electrodes showing a significant difference between the average ITC over the time window of interest in the 6 Hz (positive one-tailed test) and 15 Hz (negative one-tailed test) frequency range was higher than would be expected by chance (p_corr_ < 0.05 considered statistically significant)^36,37^. For the recognition phase only hit trials from each entrainment condition were included in the statistical analyses.

In addition to the statistical testing used by Wimber *et al*.^17^ we carried out cluster-based random permutation tests^20^ in order to compare these with the main statistical results, and to investigate how robust the findings are. In short, a non-parametric Wilcoxon signed-rank test was performed for every data point (electrode-time point) in the 6 Hz frequency range (positive single-tailed test) and in the 15 Hz frequency range (negative single-tailed test) separately, comparing trials from the 6 and 15 Hz entrainment conditions. A pre-set significance level was chosen (here 5% single-tailed) and any data points not exceeding this level were discarded (set to zero). Clusters were calculated from the remaining data points based on their adjacency in space (adjacent electrodes) and time. Cluster-level statistics were then calculated by summing resultant Z-values for all data points in each cluster. A permutation distribution was created by randomly assigning participant averages to one of the two conditions 3000 times, and each time calculating cluster-level statistics as just described. The highest cluster-level statistic from each randomization was entered into the permutation distribution and the cluster-level statistics calculated for the measured data were compared against this distribution (cluster corrected p < 0.05 considered significant).

### Data availability

The datasets generated during and/or analysed during the current study are available from the corresponding author on reasonable request.

## Electronic supplementary material

Supplementary Information
