# Supplementary material for: Assessing the utility of frequency tagging for tracking memory-based reactivation of word representations
Source: Sci Rep. 2018 May 21;8:7897. doi: 10.1038/s41598-018-26091-3 (PMC5962640; doi:10.1038/s41598-018-26091-3)
Supplement: Supplementary file 1 — Supplementary Information [file 41598_2018_26091_MOESM1_ESM.docx]

# Supplementary Information

# Assessing the utility of frequency tagging for tracking

# memory-based reactivation of word representations

*Ashley Glen Lewis*^a,b,c^, Herbert Schriefers^d^, Marcel Bastiaansen^e,f^ & Jan-Mathijs Schoffelen^b^*

^a^ Haskins Laboratories, 300 George Street, New Haven, CT 06510, USA

^b^ Radboud University, Donders Institute for Brain, Cognition and Behaviour, Centre for Cognitive Neuroimaging, Nijmegen, the Netherlands

^c^ Neurobiology of Language Department, Max Planck Institute for Psycholinguistics, Nijmegen, the Netherlands

^d^ Radboud University, Donders Institute for Brain, Cognition and Behaviour, Centre for Cognition, Nijmegen, the Netherlands

^e^ Tilburg University, Department of Cognitive Neuropsychology, Tilburg, the Netherlands

^f^ NHTV University of Applied Sciences, Academy for Leisure, Breda, the Netherlands

Corresponding author:

Dr. Ashley Lewis

Haskins Laboratories

300 George Street, Suite 900, New Haven, CT 06510, USA

Phone: +1 203 800 1942

Email: [lewis@haskins.yale.edu](mailto:stashly@gmail.com)

**Supplementary Information**

*ITC Bias*

In the main text we raise the possibility that baseline ITC values may be biased due to unbalanced trial numbers in each entrainment condition *within* participants, and that this may obscure potential reinstatement effects in the recognition phase of our experiment. In order to address this possibility, we ran the same analysis after equating trial numbers in the 6 and 15 Hz entrainment conditions. For each participant, a random subset of trials from the entrainment condition with the higher number of hit trials (as in the main analysis, only hit trials were analysed) was selected prior to the computation of ITC values. ITC was then computed for this condition as described in the main text but based only on this subset of trials. To minimize potential bias due to this subsampling approach, the procedure was performed 100 times for each participant, and the ITC value for this entrainment condition that was entered into the statistical analyses was the mean ITC values over these 100 subsampling iterations. All other methodological details were the same as for the main analysis and are described in the main text (see *Methods*).

*Time-frequency Precision Trade-off*

In the main text we raise the issue that it was unclear what temporal and spectral precision was achieved in Wimber et al. as these details were not clearly reported. In order to check whether greater temporal precision (at the expense of spectral precision) might lead to a better ability to detect relatively transient memory reinstatement effects we ran our original analyses with a sliding window of 500 ms (instead of 1000 ms). Time-resolved Fourier spectra of the data between 2 and 22 Hz were computed using sliding windows of 500 ms applied in frequency steps of 1 Hz and time steps of 20 ms (from 0 to 2000 ms relative to target word onset). Each window was tapered using a Hanning taper, to reduce spectral leakage. This resulted in a frequency resolution of 2 Hz, while the estimate at each time point is averaged data from the preceding and following 250 ms. All other details of how ITC was computed were identical to those described in the main text (see *Methods*).

Reinstatement was again present in the 15 Hz entrainment condition in roughly the same time interval as was initially observed (between 780 and 1000 ms relative to word onset). Words from the 15 Hz entrainment condition exhibited stronger phase locking at 15 Hz than words from the 6 Hz entrainment condition (p_corr_ = 0.002) at 12 electrodes (Figure S1). Similarly, the marginal effect in the 6 Hz entrainment condition was again observed between 520 and 620 ms relative to word onset. There was a marginally significant difference in phase locking at 6 Hz between words from the 6 Hz and the 15 Hz entrainment conditions (p_corr_ = 0.08) at 7 electrodes (Figure S1). The cluster-based approach for multiple-comparison correction again yielded no statistically significant differences between the two entrainment conditions at either 6 Hz (p_corr_ = 0.914) or at 15 Hz (p_corr_ = 0.112).

**
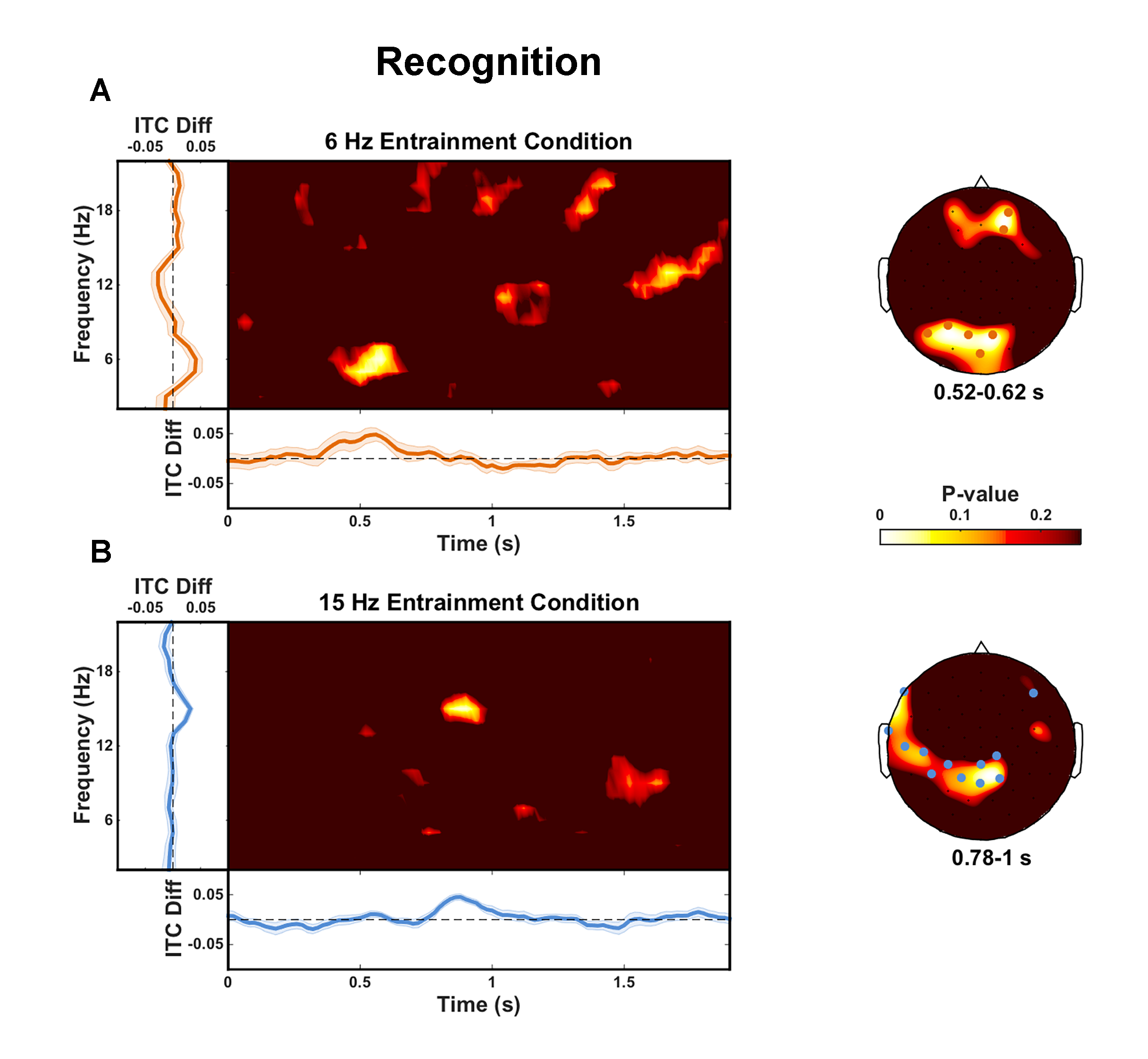
**

**Supplementary Figure S1.** ***ITC differences during recognition: 500 ms sliding window.***

ITC difference between correctly recognized target words from the 6 compared to the 15 Hz entrainment condition (A) and for correctly recognized target words from the 15 compared to the 6 Hz entrainment condition (B). Greater phase consistency (marginally significant) was present at 6 Hz for words from the 6 Hz entrainment condition (520 to 620 ms; 7 electrodes). Greater phase consistency was present at 15 Hz for words from the 15 Hz entrainment condition (780 to 1000 ms; 12 electrodes). In all panels, time-frequency plots show uncorrected P-values averaged over all electrodes exhibiting a statistically significant entrainment effect; line plots to the left show average ITC differences over the significant time interval and significant electrodes as a function of frequency; line plots at the bottom show average ITC differences over significant electrodes at the frequency of interest as a function of time; shaded regions indicate standard error of the mean; scalp plots (panels A and B) show uncorrected P-values averaged over the time interval of interest for the frequency of interest; electrodes showing differences over the entire time interval of interest are marked (orange = 6 Hz entrainment condition, blue = 15 Hz entrainment condition).
